# Capillary Hemangioma in Maxillary Anterior Region: A Case Report

**DOI:** 10.5005/jp-journals-10005-1253

**Published:** 2014-08-29

**Authors:** V Satish, Manohar Bhat, Prabhadevi C Maganur, Parth Shah, Vijay Biradar

**Affiliations:** Profssor, Department of Pediatric Dentistry, Jaipur Dental College Jaipur, Rajasthan, India; Pofessor and Head, Department of Pediatric Dentistry, Jaipur Dental College Jaipur, Rajasthan, India; Reader, Department of Pediatric Dentistry, Jaipur Dental College Jaipur, Rajasthan, India; Ex-Postgraduate Student, Department of Pediatric Dentistry, Jaipur Dental College Jaipur, Rajasthan, India; Reader, Department of Oral Pathology, Jaipur Dental College, Jaipur Rajasthan, India

**Keywords:** Hemangioma, Capillary hemangioma, Benign tumor, Vascular lesions

## Abstract

Hemangiomas are relatively common benign proliferative lesion of vascular tissue origin. They are often present at birth and may become more apparent throughout life. They are seen on facial skin, tongue, lips, buccal mucosa and palate as well as muscles. Hemangiomas occur more common in females than males. This case report presents a case of capillary hemangioma in maxillary anterior region in a 10-year-old boy.

**How to cite this article:** Satish V, Bhat M, Maganur PC, Shah P, Biradar V. Capillary Hemangioma in Maxillary Anterior Region: A Case Report. Int J Clin Pediatr Dent 2014;7(2):144-147.

## INTRODUCTION

Hemangioma is relatively common benign proliferative lesion of vascular tissue origin, which may be present either at birth or may arise during early childhood.^1^ Hemangiomas are considered to be benign tumors of infancy that are characterized by a raid growth phase with endothelial cell proliferation followed by gradual involution.^2^ The proliferation of the cells does not undergo malignant transformation.^3,4^ Most hemangiomas cannot be recognized at birth, but arise subsequently during the 8 weeks of life, but older age individuals may also be occasionally be affected. Incidence of occurrence is seen in female than male.^2^ Eighty percent of hemangioma occurs as single lesion, but 20% of affected patients will have multiple tumors.^2^ They appear as pale macules with thread-like telangiectasia over the skin and mucous membrane.^1^ They are soft and painless. They are described clinically as a soft tissue mass, smooth or lobulated sessile or pedunculated with variable size. They may be smooth or irregular bulbous in outline.^5^ This article describes the case of a 10-year-old boy reported with a growth arising from attached gingiva in the maxillary anterior region.

## CASE REPORT

**Fig. 1:**
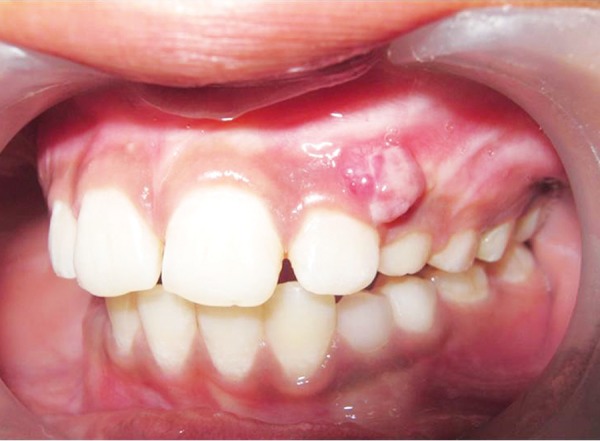
Preoperative image showing a gingival soft tissue overgrowth irt 22 and 63

A male patient aged 10 years reported with a painless swelling in upper left anterior region from past 3 months. Soft tissue overgrowth was seen in relation to 22 and 63 involving attached gingiva (Fig. 1). History revealed excision of the lesion 3 months back in the same region. After 1 month of excision, the lesion reoccurred. Patient does not give any history of pain in that region.

## GENERAL EXAMINATION

Extraoral examination including the lymph nodes was insignificant. Intraoral examination revealed a localized gingival growth which was pinkish white in color, arising from the attached gingiva of deciduous maxillary canine and maxillary 1st molar but was not interfering the occlusion. Tooth no. 64 was having proximal caries. There was grade II mobility with respect to 63 and 64. The oral hygiene was reasonably good.

**Fig. 2:**
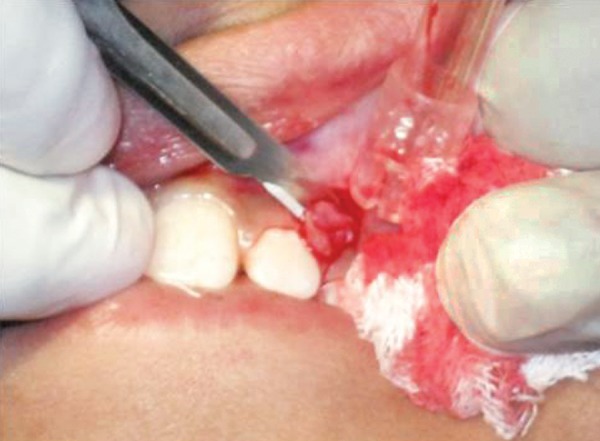
Incision with no. 15 BP blade

**Fig. 3:**
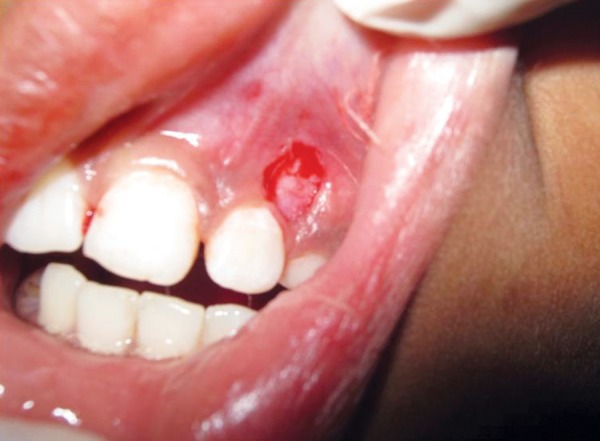
Complete excision of the lesion

**Fig. 4:**
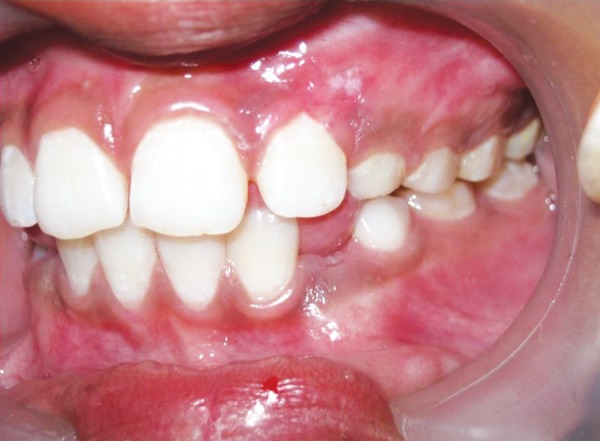
Postoperative image – after 1 month

**Fig. 5:**
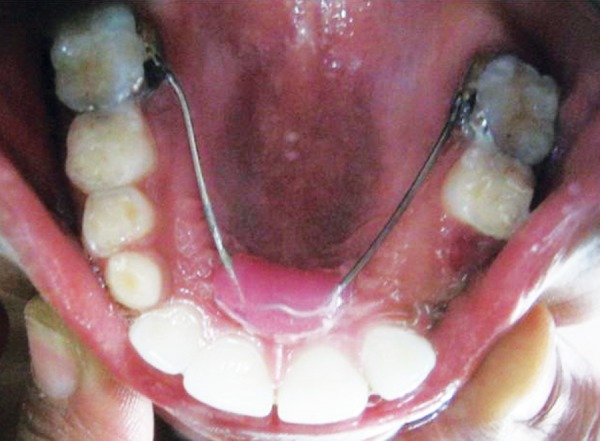
Extraction of 63 and 64 followed by Nance palatal arch space maintainer

**Fig. 6:**
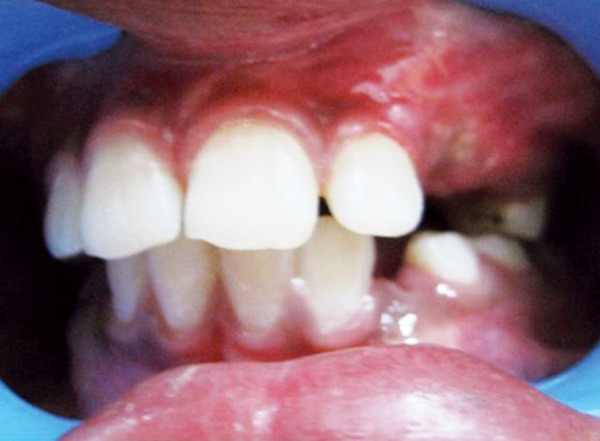
Follow-up after 2 months

A provisional diagnosis of hemangioma was made due to its clinical appearance and its growth. Preoperative hematological test revealed all findings to be normal. The lesion was excised under local anesthesia (Figs 2 and 3). Postsurgical instructions were given. The excised growth was sent to histopathological examination. Satisfactory uneventful healing occurred after 1 month (Fig. 4). Tooth no. 63 and 64 were extracted and Nance palatal arch space maintainer was delivered (Fig. 5). Patient is under follow-up to monitor the reoccurrence (Fig. 6).

### Description of the excised lesion

Single mass of tissue measuring 0.8 × 0.5 × 0.1 cm, pinkish white in color, firm in consistency, roughed surface and irregular in shape.

## MICROSCOPIC FEATURES

The H&E stained soft tissue section shows lobulated cellular growth which is separated by fibrillar connective tissue. These lobules contain plump proliferating endothelial cells, combination of numerous well and poorly canalized blood vessels which are lined by endothelial cells.

**Fig. 7:**
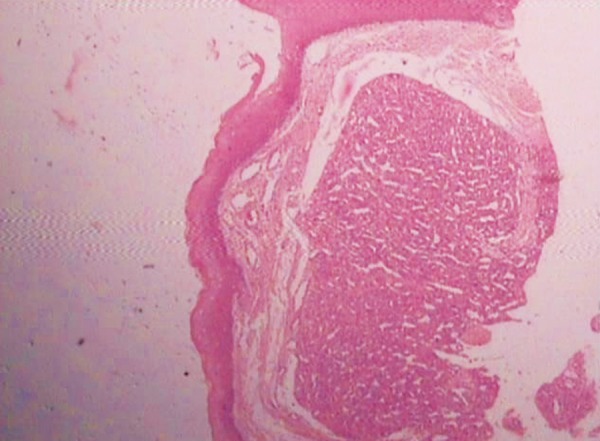
Complete area of epithelium and connective tissue filled with capillaries (4× view)

**Fig. 8:**
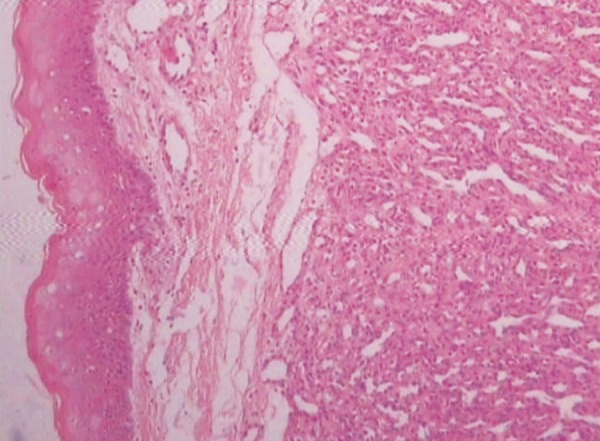
Epithelium and collagenous stroma showing capillaries (10× view)

**Fig. 9:**
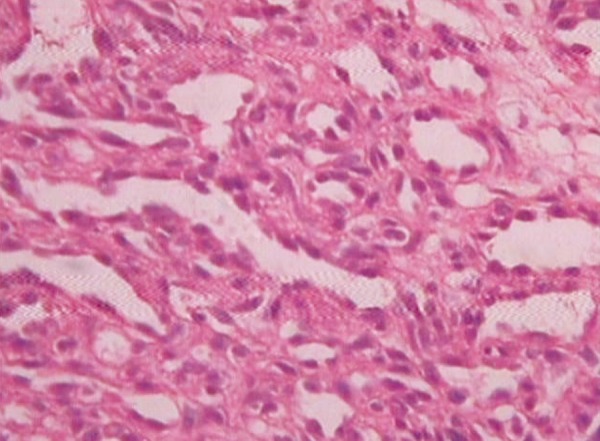
Small to enlarged capillaries lined by endothelial cells (40× view)

The overlying epithelium is parakeratotic stratified squamous type. The intervening connective tissue stroma is fibrillar composed of loose bundles of collagen fibers. It is sparsely infltrated with chronic inflammatory cells predominantly lymphocytes and plasma cells (Figs 7 to 9).

## DIAGNOSIS

On the basis of clinical and histopathological findings capillary hemangioma was made.

## DISCUSSION

The word hemangioma comes from Greek word, hema – ‘blood’, angeio – ‘vessel’, oma – ‘tumor’. This terminology has been widely used in medical and dental literature. In 80% of cases, hemangiomas occur as single lesions. More over, capillary hemangiomas have a 3:1 female to male ratio and occur more frequently among Caucasians than other racial groups.^6^ Although the exact etiology is unknown, some authors believe that this lesion is not true lesion, but rather a developmental anomaly or hamartoma.^7^

Vascular lesions are the most common congenital abnormality.^8,9^ Vascular lesions are generally divided into two categories: Hemangioma and vascular malformations. Hemangiomas are benign vascular anomalies characterized by benign proliferation of blood vessels. There are no welldefined criteria for the diagnosis and treatment of oral capillary hemangioma.^10^ The term hemangioma has been commonly used to describe a large number of vasoformative tumors. They are benign, vascular tumor that can lead to disfigurement or life-threatening consequences. Hemangiomas are usually classified into capillary, cavernous and mixed hemangioma.

**Table:** 

*Capillary hemangioma*		*Cavernous hemangioma*	
Numerous proliferating small thin-walled blood filled vessels		Deep irregular, dermal tangles of large thin-walled vessels or sinusoids	
Surrounded by discontinuous layer of pericytes and reticular fibers		Surrounded by discontinuous layer of endothelial cells	
Single layer of fattened or plump endothelial cells		Separated by scanty connective tissue	

The diagnosis of hemangioma is straightforward from the history and clinical examination. Hemangiomas usually appear within a few weeks after birth and have a growth rate that exceeds the growth rate of children.^11,12^ Hemangiomas generally do not affect adjacent bones and they can be shallow or deep. Sometimes, they can even involve all layers of the skin and offend the muscles.^13^

At the cellular level, hemangiomas are characterized by increased birth and death rate of endothelial cells and proliferation of mastocytes during the postnatal proliferative phase in the lesion. Derived from young proliferating hemangiomas, capillary endothelium is easily grown in cell culture mediums and forms tubes. In accumulated hemangiomas, the number of mastocytes decreases and becomes equal to natural tissues. Hence, a normal hemangioma is an endothelial tumor with a very complex life cycle of cell proliferation and natural regression. Vascular malformations are the second major group of vascular lesions. In fact, they are an abnormal morphogenesis of blood or lymphatic vessels. Vascular malformations are present at birth. However, their clinical manifestations are not obvious sometimes until late infancy or even childhood.^14^

The literature suggests capillary hemangioma is observed as a relatively rare and uncommon pathologic entity in the oral cavity, but is a common soft tissue tumor of head and neck. Occurrence of capillary hemangioma with its primary location on gingiva seems extremely rare. The most frequent site of occurrence of intraoral hemangiomas are lips, tongue, buccal mucosa and palate.^1,15^ It is very rare to find hemangioma on attached gingiva. The same observation was seen by Mishra MB et al in a 30-year-old female patient.^16^ Our case describes an capillary hemangioma in a male child occurring on the attached gingiva of maxillary anterior region. Clinical findings in corelation with histopathologic findings confirm our case as capillary hemangioma.

Regarding treatment, hemangiomas require no intervention. At an early age, spontaneous regression takes place. Only 10 to 20% require treatment, because of their size, location in their behavior. Different treatment procedures used in treating hemangiomas include microembolization, radiation, cryotherapy, sclerosing agents, surgical excision and recently Erbium lasers have been used. In the present case, the treatment compromised of complete surgical excision of the lesion. In the case discussed here, the patient is undergoing follow-up and no sign of recurrence has been seen.

## CONCLUSION

Any tumor, whether benign or malignant to be treated, requires early detection and diagnosis to avoid any complications at a later date. Capillary hemangiomas may be small but may cause problems in esthetics, mastication, phonetics and self-confidence in the human. There are many treatment options for hemangiomas where in surgical excision is one of the gold standard treatment regime.
